# Interactions between Myc and Mediators of Inflammation in Chronic Liver Diseases

**DOI:** 10.1155/2015/276850

**Published:** 2015-10-05

**Authors:** Ting Liu, Yu Zhou, Kwang Suk Ko, Heping Yang

**Affiliations:** ^1^Department of Gastroenterology, Xiangya Hospital Central South University, 87 Xiangya Road, Changsha, Hunan 410008, China; ^2^GI Liver Center, Keck School of Medicine, University of Southern California, Los Angeles, CA 90033, USA; ^3^Department of Nutritional Science and Food Management, College of Health Science, Ewha Womans University, Seoul 150-750, Republic of Korea; ^4^Division of Gastroenterology, Department of Medicine, Cedars-Sinai Medical Center, Davis Building 2094A, 8700 Beverly Boulevard, Los Angeles, CA 90048, USA

## Abstract

Most chronic liver diseases (CLDs) are characterized by inflammatory processes with aberrant expressions of various pro- and anti-inflammatory mediators in the liver. These mediators are the driving force of many inflammatory liver disorders, which often result in fibrosis, cirrhosis, and liver tumorigenesis. c-Myc is involved in many cellular events such as cell growth, proliferation, and differentiation. c-Myc upregulates IL-8, IL-10, TNF-*α*, and TGF-*β*, while IL-1, IL-2, IL-4, TNF-*α*, and TGF-*β* promote c-Myc expression. Their interactions play a central role in fibrosis, cirrhosis, and liver cancer. Molecular interference of their interactions offers possible therapeutic potential for CLDs. In this review, current knowledge of the molecular interactions between c-Myc and various well known inflammatory mediators is discussed.

## 1. Introduction 

Chronic liver diseases (CLDs) are an important cause of morbidity and mortality worldwide. Moreover, the burden of CLDs is projected to increase. Inflammatory cytokines are a group of important regulatory mediators involved in the development of CLDs. The development and progression of CLDs are associated with hepatitis B, hepatitis C, alcoholic liver disease, drug-induced liver disease, autoimmune liver disease, hepatocellular carcinoma (HCC), and cholangiocarcinoma (CCA).

c-Myc can be heterodimerized with Max to transactivate its target genes through binding the consensus sequence E box within the promoter region [1–3]. c-Myc has been implicated in regulating a wide variety of biological processes, including division, apoptosis, cellular growth, and angiogenesis [4, 5]. We will summarize the interaction of inflammatory mediators with c-Myc in CLDs. Although NF-*κ*B and AP-1 are not inflammatory mediators, they play key roles in the interaction of c-Myc and inflammatory mediators. We will also discuss their links with inflammatory mediators and c-Myc. Furthermore, we will discuss the relevance of inflammatory mediators and c-Myc for liver diseases and for the development of anti-CLD strategies.

## 2. Inflammatory Mediators

### 2.1. IL-1

IL-1 is an important upstream proinflammatory cytokine that affects immunity and hematopoiesis by inducing cytokine cascades. IL-1 mediates inflammation mainly by inducing a local cytokine network, enhancing inflammatory cell infiltration, and augmenting adhesion molecule expression on endothelial cells (ECs) and leukocytes [6].

IL-1*β*, one of the major agonists of IL-1, is only active in its processed, secreted form and mediates inflammation, promoting invasiveness, immunosuppression, and tumorigenesis [7]. IL-1*β* is a potent inflammatory cytokine mainly produced by macrophages. Toll-like receptors (TLRs) play a critical role in innate immune responses. IL-1*β* production requires stimulation by TLR ligands as well as a second signal such as muramyl dipeptide- (MDP-) mediated stimulation of NOD-like receptors (NLR) or P2X7 receptors [8]. IL-1*β* is involved in nonalcoholic fatty liver disease and alcoholic steatohepatitis [9–12].

Hepatic stellate cells (HSCs) are key players in fibrogenesis in chronic liver diseases. In HSCs, IL-1*β* mediates the upregulation of fibrogenic tissue inhibitor of metalloproteinase-1 (TIMP-1) and the downregulation of bone morphogenetic protein and activin membrane-bound inhibitor (BAMBI) [1]. Moreover, IL-1*β* promotes the survival of activated HSCs in mice [13]. Overexpression of IL-1*β* triggers spontaneous liver injury and fibrosis [14].

Several oncogenes, including Myc and Ras, both mediate neoplastic transformation and activate inflammatory cytokines that establish the proinvasive tumor microenvironment [15]. Myc activation in pancreatic *β* cells rapidly induces the expression and release of the proinflammatory cytokine IL-1*β*. IL-1*β* inhibition significantly inhibits and delays Myc activation of islet angiogenesis, confirming the key role of IL-1*β*. IL-1*β* is the principal Myc effector responsible for triggering rapid onset of islet angiogenesis [16]. IL-1*β* directly affects the survival and proliferation of endothelial cells and promotes the induction of other proangiogenic factors such as matrix metalloproteinases (MMPs), TGF-*β*, TNF-*α*, angiopoietin-1, IL-6, and vascular endothelial growth factor (VEGF) A [17, 18]. Myc plays an important role in the PI3K-mediated VEGF regulation in neuroblastoma (NB) cells [19]. c-Myc is essential for vasculogenesis and angiogenesis during development and tumor progression. This effect is partially associated with a requirement for c-Myc in VEGF expression. However, c-Myc is also required for the proper expression of other angiogenic factors, including angiopoietin-1 [20]. In a transgenic model of Myc-dependent carcinogenesis such as pancreatic *β* cells, IL-1*β* is both necessary and sufficient to mediate Myc-induced release of VEGF and onset of islet neoangiogenesis.

**Figure 1:**
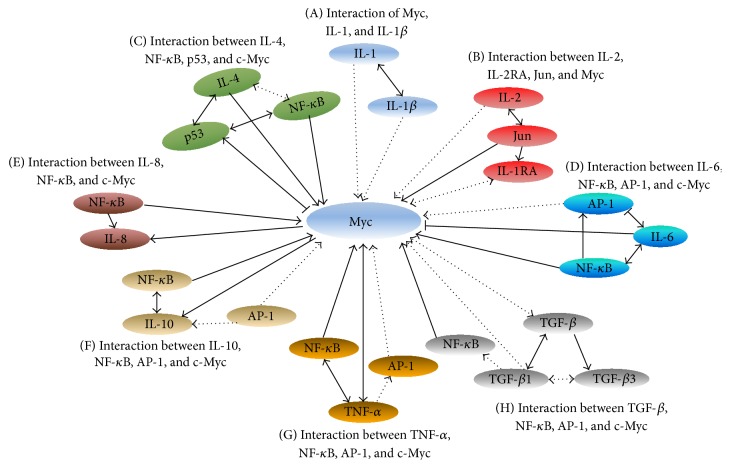
The interaction between Myc and mediators of inflammation. Arrow = positive regulation, Dot arrow = positive regulation with unclear mechanisms. Arrows in beginning and end = regulation of each other positively. Bar = negative regulation. IL-2RA: interleukin 2 (IL-2) receptor alpha. (A) Interaction of Myc, IL-1, and IL-1*β*. (B) Interaction between IL-2, IL-2RA, Jun, and Myc. (C) Interaction between IL-4, NF-*κ*B, p53, and c-Myc. (D) Interaction between IL-6, NF-*κ*B, AP-1, and c-Myc. (E) Interaction between IL-8, NF-*κ*B, and c-Myc. (F) Interaction between IL-10, NF-*κ*B, AP-1, and c-Myc. (G) Interaction between TNF-*α*, NF-*κ*B, AP-1 and c-Myc. (H) Interaction between TGF-*β*, NF-*κ*B, and c-Myc.

IL-1 expression increases in alcoholic hepatitis and cirrhosis. IL-1a expression is increased in chronic hepatitis B and hepatitis C, while IL-1*β* expression rises in alcoholic liver injury (Tables 1 and 2). IL-1*β* and IL-1 increase c-Myc expression while IL-1 increases IL-1*β* mRNA expression (Figure 1(A)).

### 2.2. IL-2

IL-2 is a pleiotropic cytokine secreted by lymphocytes that stimulates the proliferation of mucosal lymphocytes, natural killer cells, and macrophages [21]. It can also promote B cell antibody production and proliferation [22] and is essential for activation-induced cell death, important in homeostasis and eliminating potentially harmful autoreactive cells [23].

Many studies confirm that IL-2 receptors are expressed in the surface of many tumor cells, a feature that when combined with IL-2 could inhibit tumor cell growth [24, 25]. The spleen tyrosine kinase and protein tyrosine kinase (SykPTK) is physically associated with IL-2R in peripheral blood lymphocytes [26]. Therefore, SykPTK may be an integral signaling molecule engaged by the IL-2R. It has been identified that SykPTK plays a role in mediating IL-2-induced expression of c-Myc and subsequent cellular proliferation. There are two IL-2 receptor-dependent signaling pathways; one is the c-Fos/c-Jun induction pathway mediated by src family protein tyrosine kinases while the other is the c-Myc induction pathway [27]. Genistein decreases expression of rat c-Myc mRNA, which is increased by IL-2 [28]. The IL-2/IL-2R interaction causes c-Myc overexpression and cytochrome P450 (CYP) downregulation in cultured rat hepatocytes [29, 30].

IL-2 increase is associated with hepatic fibrosis in humans [31]. IL-2 directly increased c-Myc mRNA expression in rat hepatocytes and indirectly promoted c-Myc expression through activation of c-Jun in T cells from chronically infected HIV+ patients. c-Jun expression increased when bound to the AP-1 response element of a mouse c-Myc promoter. Even though IL-2 expression decreases in patients with chronic HBV and HCV infection, its expression increases in those with cirrhosis (Tables 1 and 2). IL-2 promotes c-Myc expression and a positive interaction between IL-2RA and IL-2 (Figure 1(B)).

### 2.3. IL-4

IL-4 is a multifunctional pleiotropic cytokine produced mainly by activated T cells and also by basophils, mast cells, and eosinophils, in response to receptor-mediated activation events. IL-4 plays a critical role in defining the Th2 phenotype of lymphocytes and in regulating cell proliferation, apoptosis, and the expression of numerous genes in various cell types, including macrophages, lymphocytes, fibroblasts, and epithelial and endothelial cells [32, 33].

Poly (ADP-ribose) polymerase (PARP)14 is an ADP ribosyltransferase expressed in B lymphocytes [34]. PARP14 interacts with signal transducers and activators of transcription (STAT) 6. PARP14 is required in IL-4 enhanced glycolysis in B cells, a process central to the role of PARP14 in IL-4-induced survival. PARP14 contributes to Myc-induced lymphoma pathobiology [34]. Both IL-4 and IGF-I can induce an early c-Myc response gene expression. IL-4 synergizes with IGF-I for hematopoietic cell proliferation, likely through cross talk between SHC/Grb2/MAPK and STAT6 pathways and through c-Myc gene upregulation [35]. IL-4 can promote human embryonic stem cells differentiation into “fibrogenic” fibroblast-like cells [36]. IL-4 can increase expression of c-Myc mRNA in tumor-associated macrophages and promote its translocation to the nucleus [37].

Deregulated IL-4 expression leads to direct or indirect activation of c-Myc (Figure 1(C)). Aberrant IL-4 expression is associated with HBV and HCV infection, alcoholic hepatitis, primary biliary cirrhosis (PBC), and chronic hepatitis in humans [38, 39] (Tables 1 and 2). It is interesting to characterize how IL-4-mediated c-Myc expression is involved in molecular patterns of IL-4-c-Myc, IL-4-NF-*κ*B-c-Myc, or IL-4-p53-c-Myc in CLDs.

### 2.4. IL-6

IL-6, both an immunomodulatory factor and an inflammatory mediator, could stimulate cell growth and extracellular matrix proliferation [40]. IL-6 has been identified as a central factor in liver inflammation, which leads to liver epithelial changes. IL-6 significantly increases in liver epithelia in response to stimulation and inflammatory mediators, such as endotoxin and TNF-*α* [41].

IL-6 can enhance the translation of c-Myc in multiple myeloma cells [42]. Moreover, IL-6 can promote c-Myc expression and cultured vascular smooth muscle cell proliferation [43]. The acute phase response is an inflammatory process dominated by the cytokine IL-6. STAT3 activation transduces IL-6 signaling, which induces the production of acute phase proteins such as fibrinogen and haptoglobin. IL-6 could enhance c-Myc protein expression in multiple myeloma cells independent of any effect on Myc transcription [42]. Also, IL-6 can reverse CD33 expression by upregulating Myc and subsequently downregulating CCAAT/enhancer binding protein (CEBPA) expression in myeloma cells [43].

Upregulation of human IL-6 protein is associated with infantile hepatitis syndrome, cholestasis subtype, alcoholic hepatitis, chronic hepatitis B and hepatitis C infection [29, 44, 45], cirrhosis, CCA, HCC, and experimental liver injury (Tables 1 and 2). c-Myc expression is activated by IL-6-c-Myc and IL-6-AP-1-c-Myc pathways while it is suppressed by the IL-6-NF-*κ*B-c-Myc pathway (Figure 1(D)).

### 2.5. IL-8

IL-8 is a readily activated small molecule polypeptide secreted by a variety of immune cells such as monocytes-macrophages, T lymphocytes, neutrophils, and HBV-infected liver cells. Cholangiocytes can produce IL-6, IL-8, TGF-*β*, TNF-*α*, and platelet-derived growth factor (PDGF) B chain [46, 47]. These cytokines can lead to cellular injury by stimulating an immune response and promoting tissue fibrosis. Cholangiocytes are highly responsive to Toll-like receptor (TLR) agonists [48]. Interaction of cholangiocytes with lymphocytes, HSC, and portal fibroblasts contributes to chronic inflammation and fibrosis in cholestatic liver disease [49]. c-Myc expression increases in the cholestasis-associated CCA and cholestatic liver injury [1, 2]. IL-8 is activated in patients with CLDs [50–52].

*Opisthorchis viverrini* (OV) has been reported to be an important risk factor of HCC and CCA [53]. It has been found that the secreted/excreted products of OV can induce IL-8 expression and secretion, which is a primary event in opisthorchiasis and CCA pathogenesis [54]. HBV infection can activate the immune system to induce liver cell synthesis of a large number of TNF-*α* [55], which also induces liver cells production of many IL-6 and IL-8, leading to liver inflammation and liver cell injury [56].

Hypoxia has been implicated in the pathogenesis of a broad range of liver diseases, especially in HCC and CCA [1, 53]. Gene expression regulated by hypoxia inducible factor (HIF) *α* subunits is currently very interesting due to the interaction of HIF-1*α*/HIF-2*α* and c-Myc/Max proteins. HIF-2*α* increases c-Myc activity by stabilizing the c-Myc:Max complex, which promotes cell cycle progression. However, HIF-1*α* inhibits the c-Myc function and cell proliferation [57]. Since HIF-1*α* binds to the Max protein, it competes with c-Myc and inhibits c-Myc protein stability [58]. HIF-1*α* downregulates IL-8 expression via attenuation of the Nrf2 transcription factor expression and activity in human endothelial cells [59]. Moreover, inactivation of Mxi1 (for Max interactor 1) induces IL-8 secretion activation in polycystic kidneys [60]. Nrf2 and c-Myc attenuation downregulates IL-8 expression in hypoxia [61].

IL-8 increase is associated with chronic hepatitis B and hepatitis C, alcoholic hepatitis, CCA, HCC, and experimental liver injury (Tables 1 and 2). Upregulation of human IL-8 protein in serum is also associated with human liver cirrhosis while upregulating transgenic c-Myc protein in mouse liver increases progression to hepatocarcinoma in mice [62, 63]. Upregulation of IL-8 is associated with Myc-IL-8 and NF-*κ*B-Myc-IL-8 circuitry (Figure 1(E)).

### 2.6. IL-10

IL-10 is a negative regulator mainly secreted by Th2 cells, activated B cells, monocytes, and macrophages. It helps regulate immune and inflammatory responses and tumorigenesis. IL-10 is an important anti-inflammatory mediator essential for attenuating inflammatory responses. For example, mice lacking IL-10 are more likely to die from excessive inflammatory responses when exposed to bacterial pathogens. JAK2 inhibition induced apoptosis required the inhibition of autocrine IL-10 and c-Myc expression [64]. In addition, Myc inactivation correlated with elevated levels of IL-10 receptor, causing dormancy in murine two-hit B lymphomas [65]. Transgenic c-Myc and mTOR-activated signaling increase mouse IL-10 expression in serum from mice exhibiting anorexia-cachexia [66]. IL-10 decreases regeneration of liver and inflammatory liver injury [32, 45].

The Ser/Thr protein kinase mammalian target of rapamycin (mTOR) critically regulates cell growth, proliferation, apoptosis, and metabolism. mTOR pathway inhibition downregulated renal tissue p53 expression [67]. Hypoxia induced p53, especially in the IL-10 [68]. A physical association between mTOR and the transcription factor signal transducer and activator of transcription-1 (STAT1) was recently identified in human cells, suggesting a similar role for mTOR in interferon-*γ*-stimulated gene transcription. mTOR inactivation enhanced its association with STAT1 and increased STAT1 nuclear content in PP2Ac-dependent fashion [69] while STAT1 could also regulate IL-10 and Myc.

Upregulation of IL-10 is associated with chronic HBV and HCV infection and HCC. IL-10 downregulation is related to alcoholic hepatitis (Tables 1 and 2). The interaction of IL-10 and c-Myc pathways includes Myc-IL-10, NF-*κ*B-Myc-IL-10, and AP-1-Myc-IL-10 (Figure 1(F)).

### 2.7. TNF-*α*

TNF-*α* is an adipokine involved in systemic inflammation and belongs to a group of cytokines that stimulate the acute phase reaction. TNF-*α* regulates immune and inflammatory responses, tissue remodeling, cell motility, cell cycle, and apoptosis. TNF-*α* is one of the major inflammatory mediators in liver fibrosis and a major contributor of alcoholic liver disease. TNF-*α* and its cognate receptors activate the JNK (c-Jun N-terminal kinase) pathway signaling cascade. JNK has been found to promote cell survival by regulating c-Jun and cell death by regulating c-Myc and p53 activity. Other researches [70] also found that long term elevated levels of TNF-*α* increase the tendency toward malignant transformation in mesenchymal stem cells (MSCs) through NF-*κ*B-mediated upregulation of the oncogenes c-Myc and c-Fos. Dysregulation of TNF-*α* production has been implicated in a variety of human diseases such as CLDs.

TNF-*α* regulates c-Myc expression in a cell-type specific manner. TNF-*α* treatment could markedly induce the expression of gene c-Myc and cyclin D1 in cancer cells [71]. TNF-resistant cells could overexpress c-Myc in C3H mouse embryo fibroblasts [72]. TNF-*α* has been shown to downregulate the expression of c-Myc in HL60 cells [73].

### 2.8. TGF-*β*

TGF-*β* is a pleiotropic cytokine with key roles in development, immunity, wound healing, and carcinogenesis [74]. Hepatic macrophages can produce TGF-*β*, which promotes myofibroblast fibrogenesis. TGF-*β* not only mediates its profibrotic actions by stimulating hepatic stellate cells (HSCs) through Smad-dependent pathways, but also represses HSC proliferation. HSCs also produce TGF-*β* to a lesser degree. TGF-*β* upregulation occurred in chronic HBV and HCV infection, alcoholic hepatitis, PBC, HCC, and CCA (Tables 1 and 2).

TGF-*β* could induce Myc expression by stimulating Smad3 [75]. It has been found that* Helicobacter* infection led to increased production of TNF-*α* in colonic tissue from Smad3−/− mice [76]. As mentioned above, Myc interacts with E2F1, which could be induced by Kruppel-like factor 6 (KLF6). Moreover, KLF6 could stimulate TGF-*β*1. The c-Myc expression in fibroblasts is initially repressed by TGF-*β*, but subsequent cyclin D1/cyclin-dependent kinase 4 (CDK4) goes through a complete functional change to stimulate c-Myc. TGF-*β* inhibits cell growth by downregulating c-Myc via the Smad2 phosphorylation at the C-terminal regions (pSmad2C and pSmad3C) pathways [77].

## 3. Key Players Link c-Myc and Mediators of Inflammation

### 3.1. NF-*κ*B

NF-*κ*B appears to play a major role in the network regulation of inflammatory genes and Myc. NF-*κ*B is composed of c-Rel, RelA(p65), RelB, NF-*κ*B1(p50), and NF-*κ*B2(p52). The five subunits share a conserved N-terminal domain that mediates DNA binding, dimerization, and nuclear import. It also has been found that murine c-Myc is a direct transcriptional target of Rel/NF-*κ*B, which upregulates c-Myc. B cells lacking p50 and c-Rel fail to increase in size upon mitogenic stimulation due to reduced induction in c-Myc expression. NF-*κ*B activation pathways have type 1 (p50-dependent) and type 2 (p52-dependent) pathways. While LPS and B cell activation factor (BAFF) mainly activate the type 1 or type 2 pathways, respectively, CD40 ligand (CD40L) strongly activates both [78]. c-Myc was induced in anti-CD40 and LPS treatment group. NF-*κ*B knockout in mice and c-Rel knockout in mice decrease expression of mouse c-Myc mRNA in primary B lymphocytes. NF-*κ*B increases regulation of the c-Myc promoter upstream regulatory element [79].

Peroxisome proliferator-activated receptor gamma (PPAR*γ*) expression is involved in macrophage inflammatory responses, T cell proliferation, cytokine production, and B cell proliferation as well as immune regulation. PPAR-gamma can inhibit HSC proliferation, hepatic fibrosis [80], and HCC metastases in vitro and in mice [81]. Liver-specific PPAR*γ* deficiency improves fatty liver in ob/ob mice [82]. PPAR-*κ* may be an important molecule in mediating NF-*κ*B and Myc expression. PPAR*γ* agonists activated NF-*κ*B (p50, Rel A, and c-Rel) binding to the upstream NF-*κ*B regulatory element site of c-Myc [83]. PPAR*γ* agonists increased binding of a DNA fragment containing an upstream NF-*κ*B regulatory element from c-Myc gene and mouse p50 protein [84].

P65 can mediate c-Myc expression. Using the inducible c-MycER system and c-Myc null fibroblasts found c-Myc expression significantly inhibited p65-mediated transactivation [85]. c-Myc expression inhibited NF-*κ*B activation by interfering with p65 transactivation. They also found c-Myc expression could not inhibit the transactivation potential of p65. Their studies suggest that c-Myc attenuated NF-*κ*B transcription by impairing p65 transactivation and subsequently sensitized cells to TNF-mediated apoptosis. Furthermore, c-Myc protein decreases transcriptional activator activation of human p65 increased by TNF protein. P65 and p50 can transactivate the c-Myc promoter [86]. Blocking p65 protein synthesis with specific antisense oligonucleotides greatly reduced carcinoma cell growth rate [87]. The inhibitory effect seems to be mediated by the suppression of c-Myc gene expression, since treatment with antisense oligonucleotides for p65 gene interfered negatively with c-Myc gene expression. p65 antisense decreases human c-Myc mRNA expression. NF-*κ*B/Rel transcription factors could regulate many genes including the c-Myc oncogene [87]. There is a relationship between the p65 and aryl hydrocarbon receptor (AhR) [88]. AhR and RelA increase c-Myc protein expression. This relationship activates c-Myc gene transcription in breast cancer cells. In transient cotransfection, p65 and AhR gene products demonstrated cooperation in transactivating the c-Myc promoter, which was dependent on the NF-*κ*B elements, and in inducing endogenous c-Myc protein levels. Thus, p65 participated in the expression of c-Myc gene.

It has been reported that transgenic c-Myc in mouse liver increases formation to hepatocarcinoma in mice [63, 89]. Mutant human c-Met and c-Myc also increase mouse hepatocarcinoma formation [90]. c-Myc gene knockout decreases size of hepatocytes [91] and decreases ploidy of hepatocytes in mouse liver [92]. p50/p105 knockout decreases hepatocytes proliferation in livers from mice treated with diethylnitrosamine [93]. In 129S1/Sv mouse, NF-*κ*B knockout increases liver inflammation in mice [94]. p50 knockout increases liver injury in mice, which involve* T. congolense*-variant antigen type 13 [95]. Thus, it has been discovered that both c-Myc and v-Myc can induce a truncated form of the p65, RelA(p37) [96]. More and more data demonstrate that transcriptional repression of NF-*κ*B can be mediated by c-Myc under certain physiological circumstances [97, 98].

### 3.2. AP-1

c-Myc gene overexpression is implicated in HCC in the hepadnavirus-infected woodchucks [99], ground squirrels [100], cholestasis-accelerated CCA [1], and LCA-mediated liver injury [101]. In chronic diseases, c-Myc overexpression may significantly predispose the liver to hepatocarcinogenesis [102]. In general, c-Myc promotes a cell survival unless exposed to environmental stress such as enforced c-Myc overexpression.

c-Fos, which heterodimerizes with c-Jun, leads to a more stable AP-1 complex that increases the capacity of c-Jun to transactivate target genes. c-Myc expression requires phosphorylation and nuclear translocation of extracellular signal-regulated kinase (ERK), which produces c-Fos phosphorylation and forms a specific AP-1 [103]. c-Fos downregulation in dysplastic liver nodules is associated with the initiation stage of liver cancer in humans [104]. Deletion analysis of the promoter region of the c-Fos gene indicated that the ATF2 responsive element conferred the Myc-induced expression of c-Fos [105]. Coexpression of the dominant-negative mutants of c-Fos, p38, and Rac1 blocked the Myc-mediated apoptosis [105]. Moreover, hepatitis B virus X protein (HBx) helps downregulate human c-Fos protein increased by mouse c-Myc protein. Thus, c-Fos could be a mediator of c-Myc-induced apoptosis.

The c-Jun NH2-terminal kinase (JNK) and c-Jun in the liver play an important role in growth regulation via the JNK pathway. Both c-Jun-deficient mice [106, 107] and JNK1-deficient mice [108] exhibit major defects in liver regeneration following partial hepatectomy. Furthermore, both c-Jun-deficient mice and JNK1-deficient mice were protected against the development of HCC following exposure to the carcinogen diethylnitrosamine (DEN) [108, 109]. Even though the mechanism of JNK and c-Jun signaling in the liver that contributes to regeneration and HCC is unclear, downregulation of the proliferation inhibitor p21CIP1 and upregulation of c-Myc appear to be critical factors [107, 108].

## 4. Summary

IL-1 receptor antagonist is considered an independent marker of nonalcoholic steatohepatitis in humans [110]. Since IL-1*β* levels increase in patients with alcoholic liver disease (ALD), further studies should focus on defining regulatory mechanisms in which IL-1, IL-1*β*, and c-Myc on various cell types affect multiple cellular responses in ALD.

Elevated circulating soluble IL-2 receptors in patients with chronic liver diseases are associated with nonclassical monocytes [111]. This may not only improve our understanding of how IL-2 regulates c-Myc expression, but also allow us to focus therapeutic efforts on this downstream transcriptional master-c-Myc in the monocytes.

Drug-induced liver injury (DILI) can lead to significant patient morbidity and mortality [112]. IL-4 plays a prominent role in mediating toxicity. Hepatocyte culture DILI model will improve our understanding of how IL-4 regulates c-Myc expression and help to find therapeutic targets.

IL-8 levels increase in CLDs, especially in patients in end-stage cirrhosis and patients with cholestatic diseases. Intrahepatic IL-8 upregulation could be associated with neutrophil infiltration in patients with PBC [53]. Increased IL-8 levels were associated with hepatic macrophage accumulation in noncholestatic cirrhosis. Monocyte-derived macrophages from CLD patients, especially the nonclassical CD16+ subtype, displayed enhanced IL-8 secretion in vitro. Interestingly, IL-8 correlated with liver function, inflammatory cytokines, and noninvasive fibrosis markers [53]. c-Myc regulation represents a novel anti-IL-8 therapy for use in inflammatory liver disease.

IL-10 may play a dual role in controlling liver injury via proinflammatory cytokine TNF-*α* inhibition and ethanol-induced steatosis, leading to potentiating alcoholic liver injury and ameliorating alcoholic liver injury, or via the inhibition of the hepatoprotective cytokine IL-6 [113]. In fact, c-Myc may play an important role in regulating liver injury (Figure 1(F)). Adjusting c-Myc expression may provide a novel anti-IL-10 therapy for use in alcoholic liver injury.

Alcoholic hepatitis, chronic hepatitis B and hepatitis C, cirrhosis, CCA, HCC, and experimental injury of liver can increase TNF-*α* expression (Tables 1 and 2). On one hand, c-Myc promotes TNF-*α* expression, and on the other hand, TNF-*α*-NF-*κ*B-Myc-TNF-*α* and TNF-*α*-AP-1-Myc-TNF-*α* pathways activate TNF-*α* (Figure 1(G)). However, anti-TNF-*α* agents potentially cause DILI [114].

TGF-*β* is a key regulator in CLDs, contributing to all stages of disease progression from initial liver injury through inflammation and fibrosis to cirrhosis and HCC [115]. TGF-*β* interacts with multiple important pathways, such as NF-*κ*B, AP1, and c-Myc (Figure 1(F)). Since TGF-*β* expression is dominant in liver macrophages and low in HSCs, target TGF-*β* signaling should focus on the right cell type at the right time during CLD development.

The NF-*κ*B signaling pathway is particularly relevant to hepatitis (liver infection by* Helicobacter*, viral hepatitis induced by HBV and HCV), liver fibrosis, cirrhosis, and HCC. The NF-*κ*B-c-Myc signaling pathway is a potential target to develop hepatoprotective agents. Although several types of drugs including IKK inhibitors, antioxidants, selective estrogen receptor modulators (SERMs), proteasome inhibitors, and nucleic acid-based decoys have been demonstrated to interfere with NF-*κ*B activity at different levels, some of the drugs also influence c-Myc activity. The hepatoprotective agents for targeting NF-*κ*B-c-Myc molecular patterns need to be taken into consideration during development of new therapeutic regimens.

AP-1 plays an important role in the development of HCC [116]. AP-1 is involved in dietary obesity, hepatic lipid metabolism, and NAFLD [117, 118]. A selective AP-1 inhibitor T-5224 [119] has been investigated in phase II human clinical trials. Nevertheless, no effective AP-1 inhibitors have yet been approved for clinical use, especially in treating liver diseases. Identifying selective and efficacious AP-1 inhibitors serves as a viable therapeutic strategy for liver diseases.

Aberrant expression of IL-17, IL-20, IL-22, and IL-33 is found in chronic liver disease, but the interaction between the inflammatory mediators and c-Myc must accumulate. Our review will help to understand the links between hepatic inflammation mediators and c-Myc in CLDs.

## Figures and Tables

**Table 1:** 

Liver diseases	Inflammatory mediator expressions		References
Chronic hepatitis B and hepatitis C	Up	IL-1a, IL-4, IL-6, IL-8, IL-10, c-Jun, IFN-*γ*, TGF-*β*, and TNF-*α*	[120–124]
	Down	IL-2	

Cholangiocarcinoma	Up	IL-6, TGF-*β*, and TNF-*α*	[41, 125]

Alcoholic hepatitis	Up	IL-1, IL-4, IL-6, IL-8, TGF-*β*, and TNF-*α*	[126, 127]
	Down	IL-10	

Hepatocellular carcinoma	Up	IL-6, IL-8, IL-10, c-fox, c-Jun, NF-*κ*B, TGF-*β*, and TNF-*α*	[122, 128–131]

Primary biliary cirrhosis	Up	IL-1, IL-2, IL-6, IL-8, IL-10, c-fox, c-Jun, IFN-*γ*, NF-*κ*B, TGF-*β*, and TNF-*α*	[122, 129, 130, 132–135]
	Down	IL-10	

Infantile cholestatic hepatitis syndrome	Up	IL-6, TNF-*α*	[136]

Injury of liver	Up	IL-1*β*, IL-6, IL-8, and TNF-*α*	[45, 137]

**Table 2:** 

Genes	Functions	Expression in chronic liver diseases	References
IL-1	It activates T and B cells and monocytes	Up	[123, 132, 135, 138]
IL-2	It is necessary for the growth, proliferation, and differentiation of thymic-derived lymphocytes (T cells)	Up, down	[133]
IL-4	It induces secretion of Ig by B cells, pleiotropic effect on T cells	Up	[123]
IL-6	It is an important mediator of fever and of the acute phase response and stimulates thymocyte proliferation and fibroblast growth factor activity	Up	[137, 139]
IL-8	It acts as neutrophil chemotactic factor and can induce chemotaxis in target cells, primarily neutrophils, and also other granulocytes, causing them to migrate toward the site of infection	Up	[120, 128, 134]
IL-10	It stimulates proliferation of B cells, thymocytes, and mast cells, stimulates IgA production by B cells, and also enhances B cell survival	Up, down	[123, 124, 138]
Jun	It is intronless and is mapped to 1p32-p31, a chromosomal region involved in both translocations and deletions in human malignancies	Up	[124]
NF-*κ*B	Upon activation of either T or B cell receptor, it upregulates genes involved in T cell development, maturation, and proliferation	Up	[130]
TGF-*β*	It suppresses T cell growth and differentiation	Up, down	[121, 139]
TNF-*α*	It is an adipokine involved in systemic inflammation, is a member of a group of cytokines that stimulate the acute phase reaction, and is a mediator of immune functions in the regulation of immune cells	Up, down	[140]

## Abbreviations

ALD: Alcoholic liver disease
CCA: Cholangiocarcinoma
CLDs: Chronic liver diseases
ECs: Endothelial cells
HBV: Hepatitis B virus
HCC: Hepatocellular carcinoma
HCV: Hepatitis C virus
HSCs: Hepatic stellate cells
IFN: Interferon
IL: Interleukin
IL-1R: Interleukin-1 receptor
IL-1Ra: Interleukin-1 receptor antagonist
JNK: c-Jun N-terminal kinase
LPS: Lipopolysaccharide
MDP: Muramyl dipeptide
MSCs: Mesenchymal stem cells
NASH: Nonalcoholic steatohepatitis
NK: Natural killer
NLR: NOD-like receptor
NF-*κ*B: Nuclear factor *κ*B
PI3K: Phosphatidylinositol 3-kinase
PPAR*γ*: Peroxisome proliferator-activated receptor gamma
STAT: Signal transducers and activators of transcription
SykPTK: Spleen tyrosine kinase and protein tyrosine kinase
TLR: Toll-like receptor
TNF-*α*: Tumor necrosis factor *α*
VEGF: Vascular endothelial growth factor.
